# *In vivo *Molecular targeting effects of anti-Sp17- ICG-Der-02 on hepatocellular carcinoma evaluated by an optical imaging system

**DOI:** 10.1186/1756-9966-30-25

**Published:** 2011-03-03

**Authors:** Fang-qiu Li, Shi-xin Zhang, Lian-xiao An, Yue-qing Gu

**Affiliations:** 1 Laboratory of Molecular Biology, Institute of Medical Laboratory Sciences, Jinling Hospital, School of Medicine, Nanjing University, Nanjing 210002, China; 2 Department of Biomedical Engineering, School of Life Science and Technology, China Pharmaceutical University, Nanjing, 210009, China

## Abstract

**Background:**

As the expression of human sperm protein 17 (Sp17) in normal tissue is limited and the function is obscure, its aberrant expression in malignant tumors makes it to be a candidated molecular marker for tumor imaging diagnosis and targeting therapy of the diseases.The aim of this research is to evaluate the targeting effects of anti-sperm protein 17 monoclonal antibody (anti-Sp17) on cancer *in vivo *and investigate its usefulness as a reagent for molecular imaging diagnosis.

**Methods:**

Immunohistochemistry was used to identify the expression of Sp17 in a hepatocellular carcinoma cell line and tumor xenograft specimens. A near infrared fluorescence dye, ICG-Der-02, was covalently linked to anti-Sp17 for *in vivo *imaging. The immuno-activity of the anti-Sp17-ICG-Der-02 complex was tested *in vitro *by ELISA; it was then injected into tumor-bearing nude mice through the caudal vein to evaluate its tumor targeting effect by near infrared imaging system.

**Results:**

Overexpression of Sp17 on the surface of the hepatocellular carcinoma cell line SMMC-7721 was demonstrated. Anti-Sp17-ICG-Der-02 with immuno-activity was successfully synthesized. The immuno-activity and photo stability of anti-Sp17- ICG-Der-02 showed good targeting capability for Sp17 expressing tumor models (SMMC-7721) *in vivo*, and its accumulation in the tumor lasted for at least 7 days.

**Conclusions:**

Anti-Sp17 antibody targeted and accumulated in Sp17 positive tumors *in vivo*, which demonstrated its capability of serving as a diagnostic reagent.

## Introduction

Cancer remains one of the leading causes of death in the world. Despite advances in our understanding of molecular and cancer biology, the discovery of cancer biomarkers and the refinement of conventional surgical procedures, radiotherapy, and chemotherapy, the overall survival rate from cancer has not significantly improved in the past two decades [1,2]. Early noninvasive detection and characterization of solid tumors is a fundamental prerequisite for effective therapeutic intervention. Emerging molecular imaging techniques now allow recognition of early biomarker and anatomical changes before manifestation of gross pathological changes [3-6]. The development of novel approaches for *in vivo *imaging and personalized treatment of cancers is urgently needed to find cancer-specific markers, but there is still limited knowledge of suitable biomarkers.

Sperm protein 17 (Sp17) was originally reported to be expressed exclusively in the testis. Its primary function is binding to the zona pellucida and playing a critical role in successful fertilization [7]. Expression of Sp17 in malignant cells was first described by Dong et al, who found the mouse homologue of Sp17 to be highly expressed in metastatic cell lines derived from a murine model of squamous cell carcinoma but not in the nonmetastatic parental line [8]. Various researchers have demonstrated the aberrant expression of Sp17 in malignant tumors including myeloma [9], primary ovarian tumors [10,11], neuroectodermal and meningeal tumors [12], and esophageal squamous cell cancers [13]. Sp17 was found in 66% of endometrial cancers (11), and 61% of cervical cancers [14] in our previous work. As the expression of Sp17 in normal tissue is limited and its function is obscure, it is reasonable to predict that aberrant expression of Sp17 in malignant tumors could be a molecular marker for tumor imaging diagnosis and targeting therapy of the diseases.

Molecular imaging methods permit noninvasive detection of cellular and molecular events by using highly specific probes and gene reporters in living animals, some of which can be directly translated to patient studies. A novel optical imaging technique in cancer is the use of near-infrared (NIR) light (700 to 900 nm) to monitor the site and size of the cancers [15]. The fundamental advantage of imaging in the NIR range is that photon penetration into living tissue is higher because of lower photon absorption and scatter [16]. An additional advantage is that tissue emits limited intrinsic fluorescence (i.e., autofluorescence) in the 700 nm to 900 nm range. Therefore, fluorescence contrast agents that emit in the NIR range demonstrate a favorable signal-to-background ratio(SBR) when used in animal models or for patient care, especially for endoscopy. Optical imaging is a very versatile, sensitive, and powerful tool for molecular imaging in small animals.

The near infrared fluorescence dye ICG-Der-02 (indocyanine Green derivative 02) is a derivative of indocyanine green (ICG), which was approved by the FDA (Food and Drug Administration) to be used in human subjects. Compared to ICG, the self-synthesized ICG-Der-02 organic dye holds favorable hydrophilicity and higher fluorescence quantum yield with excitation and emission peaks at 780 nm and 810 nm, respectively. ICG-Der-02 offers one carboxyl functional group on the side chain which enables the dye to be covalently conjugated to the biomarker for *in vivo *optical imaging [17].

In this study, we first demonstrated the overexpression of Sp17 in the hepatocellular carcinoma cell line SMMC-7721 and in xenografts in mice. After synthesis of anti-Sp17-ICG-Der-02, we evaluated the targeting effect of anti-Sp17-ICG-Der-02 on tumors *in vivo *with a whole-body optical imaging system in animal models.

## Materials and methods

### Cell line and monoclonal antibody

The human hepatocellular carcinoma cell line SMMC-7721 expresses high levels of Sp17 and was used for *in vitro *and *in vivo *experiments, Sp17- HO8910 ovarian cancer cell line used as negative control. The cells were cultured in RPMI 1640 medium (Invitrogen) supplemented with 10% fetal bovine serum (Hyclone) in a humidified incubator maintained at 37°C with 5% CO_2 _atmosphere and medium was replaced every 3 days. The anti-human Sp17 monoclonal antibody clone 3C12 was produced in our laboratory as previously described [14]. Monoclonal antibodies were purified from hybridoma ascites using a HiTrap Protein G HP affinity column (Amersham Biosciences).

### Tumor animal models

Male athymic nude mice (6-8 wk old, 18-22 g) were housed in a pathogen-free mouse colony and provided with sterilized pellet chow and sterilized water. All experiments were performed in accordance with the guidelines of the Animal Care Committee of the hospital. SMMC-7721 cells were treated with trypsin when near confluence and harvested. Cells were pelleted by centrifugation at 1200 rpm for 5 min and resuspended in sterile culture medium, then implanted subcutaneously into the flank of the mice (2 × 10^6 ^cells per animal). The mice were subjected to optical imaging studies when the tumor volume reached 0.5~1.8 cm in diameter.

### Immunocytochemical and immunohistochemical analysis

To investigate the expression of Sp17 in the SMMC-7721 and HO8910 cell lines, cells were cultured on a coverglass and then fixed with cooled acetone. Anti-Sp17 monoclonal antibody was then added at a concentration of 2 μg/ml and incubated overnight at 4°C. The primary antibody was detected with anti-mouse IgG labeled with horseradish peroxidase (DAKO). Diaminobenzidine (DAB) substrate was added for 7 min followed by washing with deionized water and hematoxylin was applied for 1 min to counterstain the cell on slices. Then the cell slices were dehydrated via graded ethanols followed by xylene and coverslips were attached with permount. The immunocytochemical reaction turned brown and was observed using a light microscope.

Tumor tissue sections (3 μm) from mouse model were placed on glass slides, heated at 60°C for 20 min, and then deparaffinized with xylene and ethanol. For antigen retrieval, tumor specimens mounted on glass slides were immersed in preheated antigen retrieval solution (DAKO high pH solution; DAKO) for 20 min and cooled for 20 min at room temperature. After the inactivation of endogenous peroxidase, the tissue slices were treated with anti-Sp17 monoclonal antibody and unrelated monoclonal antibody (mose anti-Candida enolase) with the same protocol as immunocytochemistry.

### Synthesis of anti-Sp17-ICG-Der-02

The synthesis of the anti-Sp17-ICG-Der-02 complex was conducted in three consecutive steps: First, the dye (1 mg, 0.001 mmol) was dissolved in H_2_O (0.5 ml) and mixed with the catalysts EDC (2.90 mg, 0.015 mmol) and NHS (1.73 mg, 0.015 mmol) (GL Biochem Co. Ltd, Shanghai, China) for the activation of the carboxylic acid functional group for about 4 h at room temperature. Next, the active ICG-Der-02 solution was added dropwise to 50 μl (200 μg) anti-Sp17 solution and then stirred at 4°C for 10 h in the dark. The reaction was quenched by adding 200 μl of 5% acetic acid (HOAc). Finally, the mixture was dialyzed (molecular weight cutoff 10 kDa) against 0.1 mol/L phosphate buffer solutions (pH = 8.3) until no free dye dialyzed out. The absorption and fluorescence emission peaks of anti-Sp17-ICG-Der-02 were located at 780 nm and 835 nm, which is exact the same as the pure ICG-Der-02, indicating the conjugation had no effect on the optical properties of NIR dye. The purified Sp17-ICG-Der-02 conjugates were stored at 4°C in the dark for future use.

### ELISA for immunological activity of ICG-Der-02 labeled anti-Sp17

Recombinant human sperm protein 17 produced in our laboratory [14] at 1 μg/ml in coating buffer were added to 96-well plates (100 μl/well) and incubated overnight at 4°C. The plates were then washed with 0.05% Tween 20/PBS and blocked with 100 μl/well of 5% fetal calf serum/PBS for 1 h at 37°C. After washing, ICG-Der-02 labeled or naked anti-Sp17 (100 μl/well), serially diluted with 5% fetal calf serum/PBS, was added and the plates were incubated for 1 h at 37°C. After a third washing, 1:2000 diluted goat anti-mouse IgG labeled with horseradish peroxidase (100 μl/well) was added and the plates were incubated for 1 h at 37°C. After another washing substrate TMB solution was added to each well and the plates were incubated for 10 min at 37°C. Finally, 2 mol/L H_2_SO_4 _was added and the plates were read at 450 nm using a Benchmark microplate reader (BIO-RAD, Hercules, CA, USA).

### In vivo and in vitro NIR Imaging

In vivo NIR imaging was performed using a self-built NIR imaging system. This NIR imaging system has been introduced in detail in our previous work [18]. In brief, a helium-neon laser (1 = 765.9 nm) is defocused to provide a broad spot with even optical density, and another 808 nm laser is supplied as background light. High sensitivity CCD camera detects the reflected light, endogenously generated luminescence or fluorescence emission. An 800 nm long pass filter could blocked the laser light (765 nm) efficiently.

Nine tumor-bearing nude mice were randomly divided into two groups. The experimental group (group A, n = 5) and control group (group B, n = 4) were both administrated anti-Sp17-ICG-Der-02 and free ICG-Der-02 through caudal vein injection. The dose for each animal was 5 μg, calculated as the amount of ICG-Der-02. The subjected mouse was anesthetized in an isoflurane chamber and immobilized in a Lucite jig before whole-body imaging at predetermined intervals (1 h, 2 h, 4 h, 6 h, 1 day, 2 days, and 3 days) post-injection. Two animals from the experimental group were observed until 7 d post-injection. Other animals were killed at 1 day and 3 days post-injection, and the tumor and major organs were taken out for *ex vivo *optical imaging examinations. All fluorescence images were acquired with 1 s exposure (f/stop = 4).

## Results

### Overexpression of Sp17 in hepatocellular carcinoma cells

**Figure 1:**
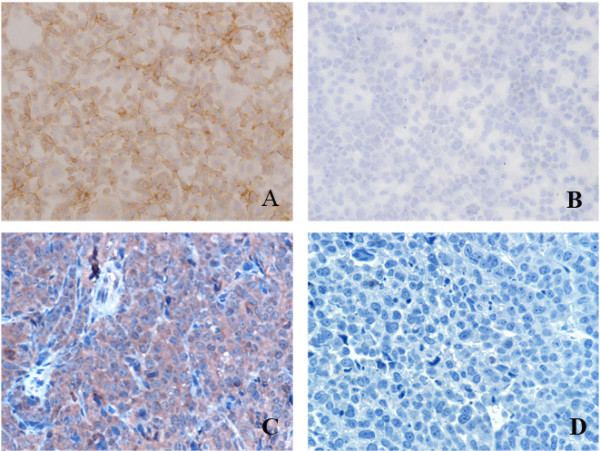
**Immunocytochemistry and immunohistochemical staining of Sp17 in a human carcinoma cell line and xenograft tumor tissues**. A, B. *In vitro *cultured cell lines staining with anti-Sp17-mAb; A: Sp17+ SMMC-7721 cells, B: Sp17- HO8910 cells (original magnification, 20×); C, D. Sp17+ SMMC-7721 cell tumor xenograft tissue slices staining with: C: anti-Sp17-mAb, D. unrelated monoclonal antibody (original magnification, 40×).

Through immunocytochemistry and immunohistochemistry, strong positive staining was observed in the human hepatocellular carcinoma cell line SMMC-7721 and its tumor xenografts tissues (Figure 1). We found Sp17 mainly localized on the cell surface of *in vitro *cultured cells and both surface and cytoplasm of xenografts tissues. This result suggested that Sp17 could be used as a marker for *in vivo *molecular imaging and targeting therapy.

### Characterization of anti-Sp17-ICG-Der-02

**Figure 2:**
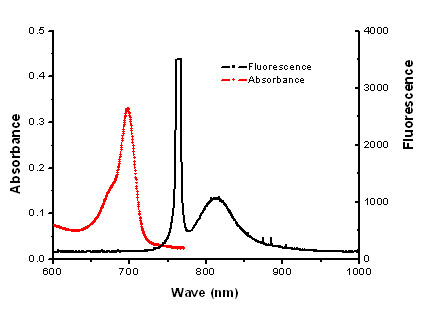
**Optical characterization of ICG-Der-02-labled anti-Sp17**.

**Figure 3:**
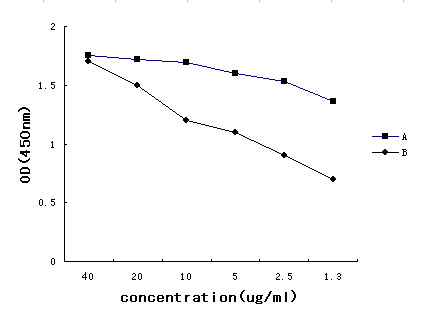
**The antibody activity of anti-Sp17-ICG-Der-02 tested with ELISA**. A. naked anti-Sp17 antibody; B. anti-Sp17-ICG-Der-02 conjugate.

The anti-Sp17 antibody was conjugated with ICG-Der-02 for *in vivo *tracing of the dynamics of anti-Sp17- ICG-Der-02 in nude mice subjects. The NHS ester of the NIR fluorescence dyes is reacted with the amino group of the amino acid residue in anti-Sp17 and purified by dialysis. The absorption and fluorescence emission spectra of the complex were characterized, as shown in Figure 2. The antibody activity of anti-Sp17-ICG-Der-02 was tested with ELISA, and the result showed that the antibody on the conjugate retained major biological activity compared with naked antibody (Figure 3).

### *In vivo *targeting capability of anti-Sp17-ICG-Der-02

**Figure 4:**
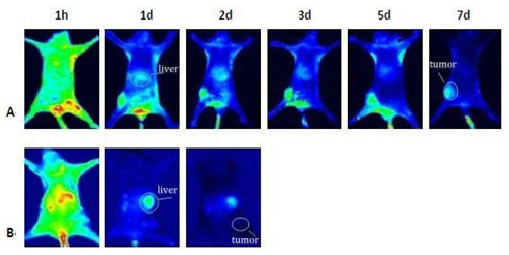
***Iv vivo *images of tumor-bearing mice show the tumor targeting effect of anti-Sp17-ICG-Der-02 (dose for each group was 0.2 μg, calculated as the amount of ICG-Der-02)**. A. Systemic injection of anti-Sp17-ICG-Der-02 (n = 5). Images were obtained in one mouse; bright fluorescent in the tumor region is due to probe accumulation. B. Systemic injection of free ICG-Der-02 (n = 3), images were obtained in one mouse, fluorescent signal in tumor is virtually absent.

**Figure 5:**
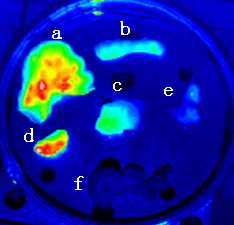
***Ex vivo *image of tumor and organs from tumor-bearing mice with systemic injection of anti-Sp17-ICG-Der-02, 1 day post-injection**. The fluorescent intensity from high to low is liver(a), kidney(d), tumor(c), spleen(b), lung(e) and colon(f).

The *in vivo *dynamic processes of anti-Sp17-ICG-Der-02 and corresponding blank samples in tumor-bearing nude mice were evaluated with an NIR fluorescence imaging system. For the experimental group, ICG-Der-02 had apparent accumulation in tumor sites at 2 h post-injection. The fluorescence intensity in the region of interest (ROI) was persistently enhanced and reached the maximum at 24 h post-injection. Strong fluorescence was observed even at 7 days post-injection for mice in this group. Images of group B (the control group) indicated that free ICG-Der-02, without the help of anti-Sp17, had little accumulation in tumor tissue at 24 h post-injection. The targeting capability of anti-Sp17-ICG-Der-02 for tumors was observed both *in vivo *imaging and *ex vitro *imaging (Figure 4 and Figure 5) after the process of entrapment. ICG-Der-02 accumulated in the liver then cleared through urine, so the liver and kidneys showed the strongest fluorescence after injection but the intensity tapered with time. From our results, we know that free ICG-Der-02 was excreted faster than anti-Sp17-ICG-Der-02.

## Discussion

Hepatocellular carcinoma (HCC) is a challenging malignancy of global importance. It is associated with a high rate of mortality and its prevalence in the United States and Western Europe and in China is increasing [19]. Early noninvasive diagnosis is needed for interventional therapy, surgery and reviewing curative effect.

Currently, the requirements for a cell surface molecule and its ligand (antibody) to be suitable as molecular imaging and targeted therapy are stringent. It is highly desirable to find an antibody that can be used to cross-link "probe molecules" for biomarker-targeted specific binding, which can not only provide sensitive and specific imaging information in cancer patients but can also selectively deliver anticancer drugs to tumor sites.

Sp17-expressing SMMC-7721 cells were selectively detected in our study with a whole-body small-animal NIR imaging system to prospectively determine the targeting activity of anti-Sp17 monoclonal antibody. Sp17 was identified as a novel cancer-testis antigen, with overexpression in various malignancies and a low level of expression in some normal tissues (including liver) [20]. We found that Sp17 was overexpressed on the surface of the hepatocellular carcinoma cell line SMMC-7721 and retained a high level of expression in xenografts in mice; thus it could be used as a suitable marker for hepatocellular carcinoma. Sp17 is a highly immunogenic protein; more than 90% of vasectomized males develop immunity against Sp17 without any harm, suggesting that Sp17 is safe for specific antibody-armed diagnosis and therapy.

The potential use of the high-affinity probe anti-Sp17 for specific NIR imaging in *in vivo *tumor diagnosis may have advantages over the existing techniques for early diagnosis of tumors. It is a noninvasive technique for *in vivo *real-time monitoring or tracing of biological information and signals in living subjects [21,22]. In this study, anti-Sp17 antibody-based targeted *in vivo *NIR imaging was investigated using ICG-Der-2 as a tracer. *In vivo *whole-body fluorescence imaging of tumors in mice with anti-Sp17-ICG-Der-02 and free ICG-Der-02 showed that tumors within mice could be clearly differentiated from normal tissues. Particularly, 3 days after application of the high-affinity probe, the most pronounced relative fluorescence signals in the tumors compared with the free dye were observed. The results showed that anti-Sp17-ICG-Der-02 maintain both the properties of the antibody and photo stability. The anti-Sp17 mAb revealed excellent targeting effect for tumors *in vivo *without non-specific binding.

## Conclusions

This *in vivo *work demonstrates that a new high-affinity antibody identifies the presence of Sp17 expression associated with the site and size of human hepatocellular carcinoma in mice. Anti-Sp17-ICG-Der-02 targeted and accumulated in Sp17 positive tumors *in vivo*, which demonstrated its capability of serving as a diagnostic reagent.

## Abbreviations

Sp17: Sperm protein 17; NIR: Near-infrared; ICG-Der-02: Indocyanine Green derivative 02; SBR: Signal-to-background; DAB: Diaminobenzidine; EDC: 1-Ethyl-3-(3-dimethylaminopropyl) carbodiimide; NHS: N-hydroxysulfosuccinimide sodium salt; TMB: Tetramethylbenzidine; ROI: Region of interest; PBS: Phosphate-buffered saline; FCS: fetal calf serum.

## Competing interests

The authors declare that they have no competing interests.

## Authors' contributions

FQL conceived, coordinated and designed the study, and contributed to the acquisition, analysis and interpretation of data and drafted the manuscript. SXZ and XLA performed the experiment and involved in drafting the article. YQG synthesized anti-Sp17-MPAICG-Der-02 and involved in drafting the article. All of the authors have read and approved the final manuscript.
